# Body mass index and altered lipid profile as major risk markers for breast cancer progression: a cross-sectional study of postmenopausal women in Pakistan

**DOI:** 10.1186/s12905-024-02929-z

**Published:** 2024-02-04

**Authors:** Lubna Gohar, Bushra Riaz, Muhammad Sohaib Nadeem, Seyyedha Abbas, Tayyaba Afsar, Suhail Razak, Fatima Muccee, Fohad Mabood Husain, Huma Shafique

**Affiliations:** 1 Army Medical College, National University of Medical Sciences, Rawalpindi, Pakistan; 2 Department of Physiology, Pakistan International Medical College, Peshawar, Pakistan; 3 Department of Clinical and Radiation Oncology, Combined Military Hospital, Rawalpindi, Pakistan; 4 Department of Biochemistry, National University of Science and Technology, Islamabad, Pakistan; 5 Department of Community Health Sciences, College of Applied Medical Sciences, King Saud University, Riyadh, Saudi Arabia; 6 School of biochemistry and Biotechnology (SBB), University of Punjab, Lahore, Pakistan; 7 Department of Food Science and Nutrition, College of Food and Agriculture Sciences, King Saud University, Riyadh, Saudi Arabia; 8 Institute of Cellular Medicine, Newcastle University Medical School, Newcastle University, Upon Tyne, UK

**Keywords:** Breast carcinoma, Body mass index (BMI), Obesity, Lipid profile, Post-menopausal

## Abstract

**Background:**

In Pakistan, the death rate for post-menopausal women with breast cancer is significant due to late detection and delayed referral to proper facilities. There are a few reports on Pakistan’s epidemiology and breast cancer risk factors. There are modifiable and non-modifiable risk factors associated with the development of breast carcinoma; of which body mass index (BMI), central obesity, and lipid profile are considered as major risk markers.

**Methods:**

This was a cross-sectional analytical study. A total of 384 women constituted the present study sample. Purposive sampling was used to collect 192 confirmed new breast cancer cases throughout the study. By using basic random sampling, an equal number of controls were chosen. Studied parameters included age, fasting blood sugar, cholesterol, triglyceride, serum high-density lipoprotein, cholesterol, serum low-density lipoprotein cholesterol, weight, height, BMI, waist circumference, and waist-to-hip ratio. The inclusion criteria of this study were post-menopausal women (45–65 years) in Pakistan. The confirmation of breast carcinoma was done through histopathology. Breast cancer occurrence was taken as a dependent variable, whereas BMI, central obesity, and lipid profile were taken as independent variables.

**Results:**

Studied risk factors (cholesterol, BMI, and central obesity) significantly correlated with breast cancer. Cholesterol has a significantly high positive correlation (0.646) with breast cancer. BMI has a positive significant correlation (0.491) with breast cancer, and central obesity has a low but positive significant correlation (0.266) with breast cancer. Moreover, the binary logistic regression model also showed a significant association between biochemical factors and breast cancer occurrence. Regression analysis depicted a linear relationship between a dependent variable (breast cancer occurrence) and independent variables (central obesity, cholesterol, BMI).

**Conclusion:**

Postmenopausal overweight (central obesity), increased BMI and high cholesterol levels are major risk factors for breast cancer. Moreover, high total cholesterol proved to be the most significant risk marker for the occurrence of breast cancer in post-menopausal women of Pakistan.

## Background

Breast cancer is the most prevalent cancer to be diagnosed and the main cause of cancer-related deaths in women around the world [1]. In Pakistani women, 2.1 million new cases and 627,000 fatalities were reported in 2018 [2, 3]. Pakistani women have a high occurrence of breast cancer among Asian countries [4]. 11% of women are at risk of receiving a diagnosis of breast cancer during their lifetime [5]. The evaluation of risk factors of breast cancer in Pakistani women has been delayed due to a lack of resources for research, a shortage of top oncologists, improper reporting of case incidence, late detection, and delayed referral to proper facilities. Hence, the death rate for post-menopausal women with breast cancer is striking [6]. Breast cancer is considerably influenced by lifestyle factors such as excessive fast food consumption, family history, and reproductive and estrogen variables, along with age. Managing these risk factors is decisive in precluding breast cancer occurrence [7].

There are modifiable and non-modifiable risk factors associated with the development of breast carcinoma [8]. Interestingly, studies indicated that premenopausal obesity defends against breast cancer, however postmenopausal obesity intensifies the risk [9]. Obesity is a most pressing global health issue and a modifiable risk factor linked to higher incidence and severity of breast cancer, particularly hormone receptor-positive breast carcinoma [10, 11]. Obese women display higher breast cancer indices and tumor sizes than normal-weight women [12]. Additionally, it has been hypothesized that having more metastatic axillary nodes at the time of diagnosis is related to obesity [13].

Furthermore, recent investigations propose that fat distribution, rather than total body fat, is a more important measure of the health risks related to obesity and breast carcinoma [14]. The risk of breast cancer is increased by high body mass index (BMI) [13], as well as the likelihood of more aggressive tumors and a worse prognosis. The adipose micro-environment in obese people shares many similarities with the tumor micro-environment such as low-grade inflammation, cellular composition, and raised levels of reactive oxygen species. In addition, the role of adipose tissue as an endocrine organ and association of adipokines secreted from the adipose tissue, in the development of breast carcinoma is vastly being studied [15].

Environmental factors, genetics, age, weight, and food all contribute to breast cancer development [16]. The relationship between weight, food, and plasma lipids/lipoproteins has been recently identified [17]. Experimental mice models have shown the importance of cholesterol and its transporters in the emergence of breast cancer [18]. Breast cancer cells that are estrogen receptor-positive have been found to proliferate and facilitate metastasis when subjected to the cholesterol metabolite 27-hydroxycholesterol [19]. Inhibiting apoptosis and enhancing the proliferation and migration of cancer cells may be caused by the activation of several inflammatory pathways by lipoprotein oxidation and HDL glycation [20]. Potential therapeutic strategies to reduce excessive cholesterol and prevent breast cancer include apolipoprotein A-I mimics and cholesterol-lowering drugs [21]. As a result, lipid-lowering medications offer hope to the millions of women whose changed lipid levels put them at risk for breast cancer. Total cholesterol was identified as a risk factor for breast cancer in a human investigation [22]. The amount of HDL-C and cancer risk are also inversely correlated in breast cancer patients. Lipoproteins can be used as markers to track the development of cancer by revealing their metabolic properties. Both clinical studies and experimental studies indicated the role of altered LDL cholesterol (LDL-C), oxidized low-density lipoprotein cholesterol (OXLDL-C), very low-density lipoprotein cholesterol (VLDL-C), and high-density lipoprotein cholesterol (HDL-C) with carcinogenesis [23]. Earlier Studies on breast cancer collectively addressed all or majority of the risk factors and their association with breast cancer. In contrast, this study, however, focuses only on the biochemical and physical parameters of obesity and their association with breast cancer in postmenopausal Pakistani women. Postmenopausal breast cancer is the most prevalent type of breast cancer among females and obesity, being a modifiable risk factor in the majority of the cases makes this study unique.

We aim to analyze the association between BMI, central obesity, and lipid profile with breast cancer in post-menopausal status of Pakistani women. Public awareness regarding these modifiable yet very strong risk factors for breast cancer can reduce the burden of this life-threatening disease.

## Methods

### Trial design and setting

This cross-sectional analytical study was carried out the at Centre for Research and Analytical Medicine (CREAM), Army Medical College, National University of Medical Sciences, Rawalpindi, Pakistan, and in collaboration with Armed Forces Institute of Pathology (AFIP), Rawalpindi, Pakistan. This study was completed in one-year duration (2020–2021).

### Sample size and sample collection

The blood samples (3 ml) were drawn from the antecubital vein on the forearm (Venous blood) at the Armed Forces Institute of Pathology (AFIP), Rawalpindi, Pakistan. Purposive sampling & random sampling techniques were deployed to collect the data from the breast cancer group and control group respectively. The inclusion criteria of this study were post-menopausal women (45–65 years) in Pakistan.

The total female population in Pakistan of the age group 45–65 years is 11,560,608 for the year 2020–2021 [27]. The age-specific breast cancer population of post-menopausal women (45–65 years) is 1,644,253 in Pakistan for the year 2020–2021 [28]. Krejice and Morgan’s table was used for sample calculation. A total of 384 women constituted the present study sample. Purposive sampling was used to collect 192 confirmed breast cancer cases throughout the study. By using basic random sampling, an equal number of controls were chosen. Data included age, blood sugar fasting (BSF), cholesterol, triglyceride, high-density lipoprotein (HDL), low-density lipoprotein (LDL), weight, height, BMI, waist circumference (WC), and waist-to-hip ratio (WHR). Data was also converted to binary data (0, 1) according to the Cut-off values mentioned above for analysis. breast cancer occurrence was taken as a dependent variable, whereas BMI, central obesity, and lipid profile were taken as independent variables.

### Sample processing

Confirmation of breast carcinoma was done through histopathology by Armed Forces Institute of Pathology (AFIP), Rawalpindi, Pakistan. Data was collected from confirmed breast carcinoma patients. Post-menopausal women of age group 45–65 years with 365 + days since the last menstrual period, with no other inflammatory or malignant disease, were included in the study. Age-matched healthy women were recruited from different social setups to add them to the control group their physical examination, completion of checklist (attached proforma) and interview were taken. Then blood samples of both groups were taken. The serum was collected from blood after centrifugation at 10,000 rpm for 15 min at 4^0^C. The details of sample processing and cut-off value are mentioned below:

Serum triglycerides were measured a using Randox triglycerides kit (NCEP-ATP III cut-off value ≥ 1.7mmol/l), and serum total cholesterol was measured using Randox UK kits (The normal measuring value at AFIP ˂ 6.2 mmol/l), serum high-density lipoprotein cholesterol was measured using (HDLC) Randox HDL-cholesterol assay kit, UK (The normal value at AFIP ≥ 1.3 mmol/l), serum low density lipoprotein cholesterol (LDLC) was measured using Randox UK kit (The normal measuring value at AFIP ˂ 2.59 mmol/l).

### Statistical analysis

A two-tailed p-value < 0.05 was considered statistically significant. Data was analyzed by using SPSS version 28. The categorical data was analyzed statistically using a prevalence ratio (PR) with a 95% confidence interval (CI). Mean and standard deviation were calculated and a t-test was applied to determine the differences between the means of the two groups (Table 1). ANOVA was applied to compare the variance within each group to the overall variance of the group means (Table 2). The prevalence ratio was calculated, and the Chi-square test was applied to see how confident we can be in claiming that our observed results deviate from our predictive outcome (Table 3). Binary logistic regression (Table 4) was applied to see progression of basic correlation.

## Results

**Fig. 1:**
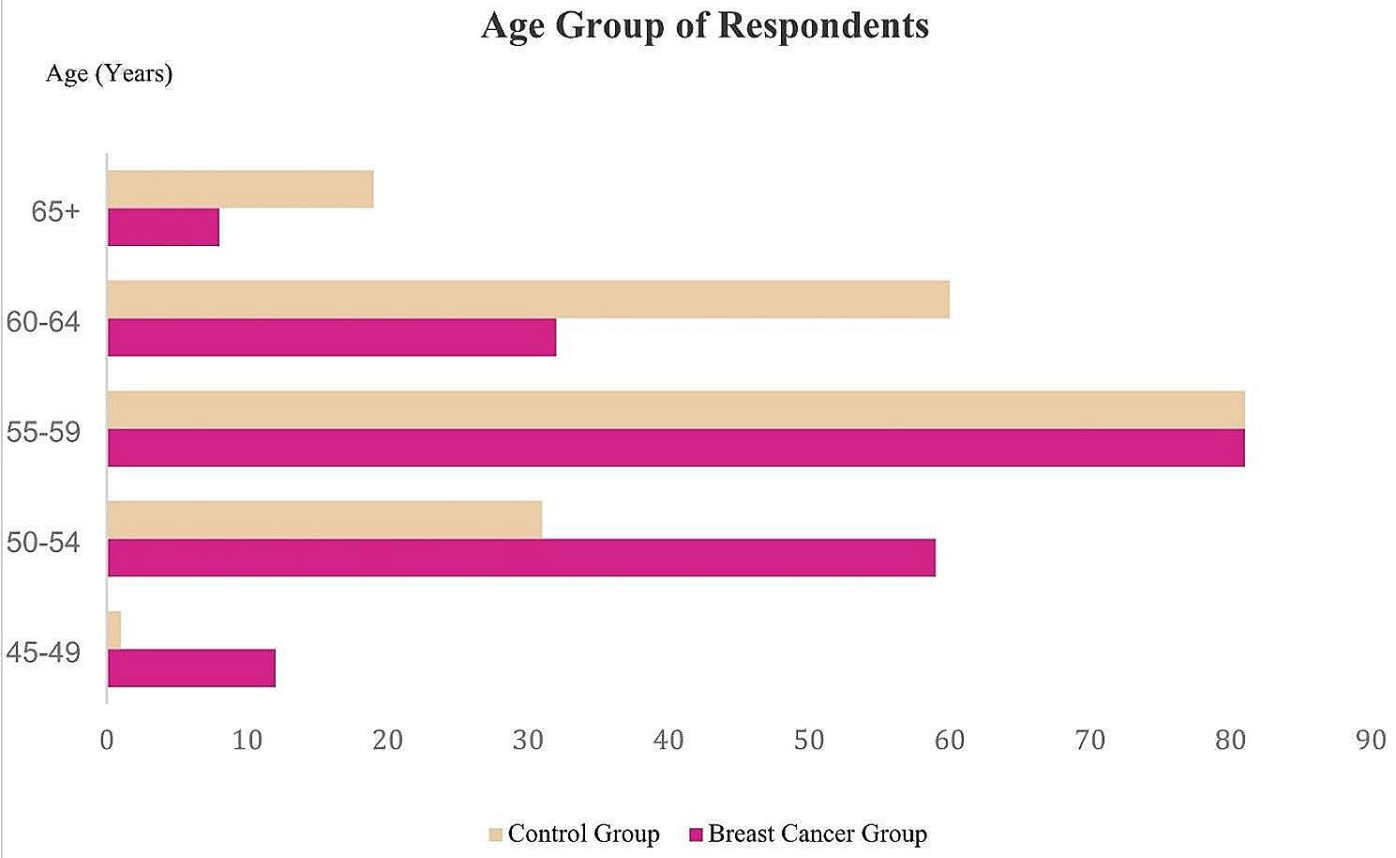
Frequency of breast cancer occurrence among various age group of respondents

Figure 1 indicates the various age groups of study participants in both control and breast cancer groups. We observed maximum breast cancer occurrence among post-menopausal women aged between 55 and 59 years (42%: 81 out of 192) while 31% from age group 50–54 years and 17% from age group 60–64 years.

Estimated physical health parameters include BMI, age, weight, and height. We noticed a significant (*p* < 0.001) increase in weight and BMI. We observed a significant (*p* < 0.001) increase in LDL while the reduction in HDL levels was recorded. Variations in lipid profile among normal control and breast cancer patients are presented in Table 1. *P* < 0.05 for all biochemical parameters, hence their mean is significantly different for breast cancer and control group.

**Table 1:** Assessment of variation in lipid biomarkers, weight, height, and age factor in breast cancer patients

Biochemical Parameters	Breast Cancer Group	Control Group
Age	56.04 **±** 4.75**	58.70 **±** 4.06
BSF	7.34 **±** 3.035**	6.3 **±** 1.41
Cholesterol	5.83 **±** 0.51**	4.78 **±** 0.82
Triglyceride	2.45 **±** 0.33**	1.92 **±** 0.78
HDL	1.01 **±** 0.19**	1.27 **±** 0.33
LDL	4.37 **±** 0.31**	3.17 **±** 0.86
Weight	71.36 **±** 12.04**	66.23 **±** 6.55
Height	153.23 **±** 7.06**	158.09 **±** 6.30
BMI	30.36 **±** 4.42**	26.65 **±** 3.03
WC	94.54 **±** 12.48**	87.10 **±** 9.56
WHR	0.87 **±** 0.04**	0.84 **±** 0.04

Data presented as Mean ± standard deviation. t-test was employed to compare the significance different of each parameter between control and breast cancer patients. Asterisks** indicates *p* < 0.001. BSF: blood sugar fasting, cholesterol, triglyceride, HDL: high-density lipoprotein, LDL: low-density lipoprotein, weight, height, body mass index: BMI, WC: waist circumference, and WHR: waist-to-hip ratio.

Binary logistic regression was applied to find the association between biochemical factors and breast cancer occurrence.

Separate ANOVA was done for each independent variable; central obesity (WHR, WC), BMI, and total cholesterol. The results of all the ANOVA along with a determination of whether there is a statistically significant difference between the group means (breast cancer group and control group) are shown in Table 2. All the significance values (*p* < 0.001) are less than 0.05, and as a result, there is a statistically significant difference between the breast cancer group and the control group.

**Table 2:** The two-way ANOVA table for cholesterol, BMI, and central obesity with breast cancer

Dependent Variable	Independent Variables	Model	Sum of squares	df	Mean Square	F	p-value
**Breast cancer occurrence (Yes = 1, No = 0)**	WHR, WC	Between groups	14.012	2	7.006	32.558	< 0.001
		Within groups	81.988	381	0.215		
		Total	192	383			
	BMI	Between groups	23.149	1	23.149	121.386	< 0.001
		within groups	72.851	382	0.191		
		Total	192	383			
	Cholesterol	Between groups	40.078	1	40.078	273.77	< 0.001
		Within groups	55.922	382	0.146		
		Total	192	383			

Two-way ANOVA was applied to study the significance of various variables in breast cancer and the control group—body mass index: BMI, WC: waist circumference, and WHR: waist-to-hip ratio

For case-control studies, prevalence ratio is the measure of association. It quantifies the magnitude of parameters in exposure (breast cancer group) and control group. The study results show high total cholesterol (OR 23.84, 95% CI 13.71–41.43), high BMI (OR 9.59, 95% CI 5.86–15.66), and high BMI (OR 3.1598, 95% CI 2.03–4.90) to be independent risk factors for breast cancer (Table 3). Study findings reveal that the prevalence ratio is greater than 1 for all biochemical parameters, which means an overall positive association between biochemical parameters and developing breast cancer, and relative risk is higher for the breast cancer group as compared to the control group.

**Table 3:** Prevalence Ratio of biochemical parameters of breast cancer occurrence using binary logistic analysis

PREVALENCE RATIO							
**Biochemical parameters**	**Breast cancer group**	**%**	**Control group**	**%**	**Prevalence ratio (95% CI)**	**Chi-Square**	**p-values**
**Cholesterol**						160.312	< 0.001
Yes	170	89	47	24	23.8395 (13.7175, 41.4303)		
No	22	11	145	76			
**BMI**						92.598	< 0.001
Yes	121	63	29	15	9.5789 (5.8576, 15.6644)		
No	71	37	163	85			
**Central obesity**						27.246	< 0.001
Yes	148	77	99	52	3.1598 (2.035, 4.9050)		
No	44	23	93	48			

Chi-square test applied to study the prevalence ratio of various variables in occurrence of breast cancer: There is no linear relationship between biochemical parameters (central obesity, cholesterol, BMI) and group (occurrence or non-occurrence of breast cancer). Body mass index: BMI.

Table 4 shows regression analysis, the high BMI (≥ 30 kg/m^2^), TC (≥ 6.2 mmol/L), and central obesity (WC ≥ 80, WHR ≥ 0.85) were found to be significant predictors of breast cancer.

The null hypothesis is rejected at a 5% level of significance (F = 136.300 and *p* < 0.05). It indicates that the regression model predicts the dependent variable (group) significantly well. There was a linear relationship between biochemical parameters and the occurrence of breast cancer in women of menopausal age.

**Table 4:** Regression analysis of cholesterol, BMI and central obesity with breast cancer occurrence

Model	Sum of Squares	df	Mean Square	F	p-values
**Regression** **Residual** **Total**	49.758	3	16.586	136.300	< 0.001
	46.242	380	0.122		
	96.000	383			

a. Dependent variable: Group (control group, breast cancer group)

b. Predictors: (constant), central obesity, cholesterol, BMI

Binary logistic regression test was applied to study the linear relationship between biochemical parameters and the occurrence of breast cancer.

However, all the major risk factors (cholesterol, BMI and central obesity) significantly correlated with breast cancer (Table 3). Cholesterol has a significantly high positive correlation (0.646) with breast cancer. BMI has a positive significant correlation (0.491) with breast cancer, and central obesity has a low but positive significant correlation (0.266) with breast cancer (Table 5).

**Table 5:** Correlation analysis of cholesterol, BMI and central obesity with breast cancer occurrence

Variables		Correlation
**Breast cancer occurrence (Yes = 1, No = 0)**	Total cholesterol	0.646***
	BMI	0.491***
	Central obesity	0.266***

Asterisks *** indicates *p* < 0.0001

## Discussion

In the study, we investigated the individual and cumulative impact of the three major risk factors, BMI, central obesity, and lipid profile on the risk associated with breast cancer in post-menopausal women of Pakistan. We also carefully examined whether these three risk factors interact, and whether relationships differ according to post-menopausal status. Our study findings revealed that for all parameters there is a significant difference between the mean of the breast cancer group and the control group. Moreover, the binary logistic regression model also shows a significant association between biochemical factors (BMI, central obesity, and total cholesterol) with breast cancer occurrence. To quantify the magnitude of biochemical parameters in the breast cancer and control group, the prevalence ratio was calculated, which shows a high magnitude of biochemical factors for the breast cancer group as compared to the control group with a significant Chi-square value. To find the linear relationship between Biochemical parameters and the occurrence of breast cancer in women of post-menopausal age, univariate multinomial regression analysis was applied. It shows a linear relationship between the dependent variable (breast cancer occurrence) and independent variables (BMI, central obesity, and total cholesterol). Correlation analysis among dependent and independent variables depicts that total cholesterol has the highest positive significant correlation with the occurrence of breast cancer, BMI is second, and central obesity has a third positive correlation with the occurrence of breast cancer in post-menopausal women of Pakistan.

Recent research has shown that women who have central obesity after menopause have a higher risk of developing breast cancer. Particularly, the effects of increased % age thickness on risk specific to breast cancer vary with BMI. Post-Menopausal women who are overweight or obese 18% of them have elevated breast compactness [24]. Our findings add to the body of literature that previously examined BMI as a risk marker for breast cancer in postmenopausal women.

A thorough research on the link between diabetes mellitus and breast cancer has revealed that patients who also had breast cancer had a significantly higher risk of diabetes [25]. Our study findings also show high BSF among the breast cancer group as compared to the control group. In a study of postmenopausal women, we discovered a connection between increased impermanence following a breast cancer diagnosis and higher BMI (≥ 25) and WHR (≥ 0.80) at the time of identification. And both, a high WHR and an above-average BMI were worse than either one alone [26]. Our study findings also show high BMI, WHR, and WC among the breast cancer group as compared to the control group.

Additionally, the presence of long-term obesity in a person indicates the presence of other chronic health conditions linked to obesity or unhealthy lifestyle choices. Likely, underweight women or those who lost weight throughout the two time periods were the sickest given that breast cancer patients commonly gain weight or have additional concomitant conditions [27]. Given that WHR and BMI in this study each exhibited unique impacts on breast cancer, many biological pathways may be involved in the link between obesity and tumor growth. Irrespective of BMI, increased WHR has been associated with a higher risk of insulin resistance and hyperinsulinemia and is thought to have that effect [28].

Researchers discovered by multivariate analysis that breast cancer risk was significantly predicted by BMI, TGs, and menopause. There are several conflicting data about the relationship between dyslipidemia and the risk of breast cancer, primarily because of variations in the study population and study region. However, numerous studies have demonstrated that dyslipidemia in one or more forms raises the risk of breast cancer. The cholesterol metabolite 27-hydroxycholesterol can act as an oestrogen, promoting the growth of oestrogen receptor-positive breast cancer cells [29]. According to investigators, the metabolic syndrome, which includes dyslipidemia as one of its symptoms, may raise the risk of breast cancer by elevating leptin and lowering adiponectin levels in the blood [30]. Research has also shown that these two hormones enhance the likelihood of developing breast cancer. Increased mammographic breast density and hyperlipidemia are correlated, which in turn raises the risk of breast cancer [31], which may be another significant relationship. Therefore, when formulating plans for the prevention and care of breast cancer in women, dyslipidemia or imbalance in Lipid profile should be taken into account. Our study findings add to the body of literature that previously examined the association between dyslipidemia or imbalance in Lipid profile and breast cancer.

The decision of the lipid acquisition pathway may also be influenced by additional elements, such as central obesity. According to recent research central obesity raises the incidence of breast cancer [14]. These results suggest that the degree to which a cell depends on its energy balance condition for malignant transformation may be determined by its cellular status. Therefore, these observations regarding abnormal plasma lipid profiles in cancer patients may reflect various metabolic tactics used by breast cancer cells.

## Conclusion

Postmenopausal overweight/ central obesity, BMI, and cholesterol levels are major risk factors for breast cancer. The findings of the study demonstrated a significant association between high levels of total cholesterol, BMI, and central obesity with breast cancer in post-menopausal women of Pakistan. To further understand the underlying mechanisms and importance of lipid profiles for the development of breast cancer, more research is necessary. Studying the connection between circulating lipids and molecular hallmarks in malignancies is particularly important. Additionally, it is important to evaluate the interaction between circulating lipids and tumour molecular characteristics on the development of breast cancer specifically for post-menopausal women with high BMI and central obesity.

### Limitations

As the research project was required to be completed in the stipulated time, we could only take a limited sample size. This was a cross-sectional study and biochemical and anthropometric parameters were assessed only once at the beginning of the disease. We could not do a follow-up of both cases and controls after some time. We were also unable to follow the breast carcinoma cases to see their prognosis and overall survival.

## Data Availability

All data generated or analyzed during this study are included in this article.
